# Management of Financial Assets by Older Adults With and Without Dementia or Other Cognitive Impairments

**DOI:** 10.1001/jamanetworkopen.2022.31436

**Published:** 2022-09-13

**Authors:** Jing Li, Shuqi Wang, Lauren Hersch Nicholas

**Affiliations:** 1 The Comparative Health Outcomes, Policy, and Economics Institute, Department of Pharmacy, University of Washington, Seattle; 2 JC School of Public Health and Primary Care, The Chinese University of Hong Kong, Hong Kong, China; 3 Department of Health Systems, Management & Policy, Colorado School of Public Health, Aurora; 4 Department of Economics, University of Colorado Denver; 5 Hopkins’ Economics of Alzheimer’s Disease and Services Center, Johns Hopkins University, Baltimore, Maryland

## Abstract

This cross-sectional study examines the extent to which older US adults with dementia or cognitive impairments manage their own finances, report difficulty doing so, and own risky financial assets such as stocks and loans.

## Introduction

Dementia and other cognitive impairments are prevalent among US older adults.^1^ Brain changes linked to cognitive impairment can lead to overconfidence, memory problems, and deficits in decision-making.^2,3^ These changes can have financial consequences, including missed bill payments, risky investment choices, and financial exploitation.^3,4^ To characterize the population at risk of cognitive impairment–associated asset mismanagement, we assessed the extent to which older US adults with and without cognitive impairment manage their own money, including risky assets (eg, stocks and loans).

## Methods

We conducted a cross-sectional analysis of the 2018 Health and Retirement Study (HRS), a nationally representative panel survey of US adults aged 50 years or older that contains comprehensive information on demographics, cognitive function, and financial assets. The study population included HRS respondents aged 65 years or older whose cognitive status could be ascertained from their response to the modified version of the Telephone Interview for Cognitive Status (TICS-M) or through proxy reports. We classified respondents as having no cognitive impairment, cognitively impaired nondementia (CIND), or dementia using the Langa-Weir classification method,^5^ a validated method that assigns the TICS-M and proxy assessments to cognition categories based on in-person clinical examinations (eAppendixes 1 and 2 in the Supplement). The Weill Cornell Medicine institutional review board approved the study and waived informed consent because only publicly available data were used. We followed the STROBE reporting guideline.

We conducted descriptive analyses, stratified by cognitive status, of demographic characteristics, including whether respondents managed their own finances (answered detailed questions about household finances on behalf of the household), self-reported difficulty in managing finances, and owned assets. We used HRS sample weights to characterize the noninstitutionalized study population. Analyses were performed from July 2021 to June 2022 using Stata, version 16. Two-sided *P* < .05 was considered significant.

## Results

The study population included 8786 HRS respondents, representing 50 676 933 adults aged 65 or older; 27 982 516 (55.2%) were female, and mean (SD) age was 74.2 (7.5) years. In all, 40 844 009 individuals (80.6%) were classified as having no cognitive impairment, 6 957 499 (13.7%) as having CIND, and 2 875 425 (5.7%) as having dementia (Table). Compared with those without cognitive impairment, respondents with CIND or dementia were older and more likely to identify as Black, Hispanic, or other race and ethnicity and to have less than a high school education.

**Table.** Summary Statistics for the Study Population by Cognition Status^a^

Variable	Individuals^b^			*P* value
	No cognitive impairment (n = 40 844 009)	CIND (n = 6 957 499)	Dementia (n = 2 875 425)	
Proxy respondent				
No	40 054 743 (98.1)	6 584 741 (94.6)	1 848 482 (64.3)	<.001
Yes	789 266 (1.9)	372 758 (5.4)	1 026 943 (35.7)	
TICS-M score, mean (SD)	17.2 (3.0)	9.5 (1.6)	4.3 (2.0)	<.001
Proxy assessment score, mean (SD)	1.2 (0.6)	3.9 (0.9)	9.3 (1.8)	<.001
Age, mean (SD), y	73.1 (6.4)	77.9 (9.5)	81.4 (10.2)	<.001
Sex				
Female	22 368 024 (54.8)	3 917 624 (56.3)	1 696 868 (59.0)	.17
Male	18 475 985 (45.2)	3 039 875 (43.7)	1 178 557 (41.0)	
Race				
Black	2 892 279 (7.1)	1 297 066 (18.6)	582 103 (20.2)	<.001
White	36 022 616 (88.2)	5 083 825 (73.1)	2 031 618 (70.7)	
Other^c^	1 929 114 (4.7)	576 608 (8.3)	261 704 (9.1)	
Ethnicity				
Hispanic	2 728 289 (6.7)	964 050 (13.9)	514 287 (17.9)	<.001
Not Hispanic	38 115 720 (93.3)	5 993 449 (86.1)	2 361 138 (82.1)	
Educational level				
Less than high school	2 956 338 (7.2)	2 265 329 (32.6)	1 140 236 (39.7)	<.001
High school or GED	21 166 563 (51.8)	3 613 993 (51.9)	1 313 641 (45.7)	
College and above	16 721 108 (40.9)	1 078 177 (15.5)	421 548 (14.7)	
Managing own finances				
No	11 557 575 (28.3)	1 662 700 (23.9)	724 714 (25.2)	.013
Yes	29 286 434 (71.7)	5 294 799 (76.1)	2 150 711 (74.8)	
Having difficulty managing own finances				
No	38 873 230 (95.2)	5 718 595 (82.2)	1 252 664 (43.6)	<.001
Yes	1 970 779 (4.8)	1 238 904 (17.8)	1 622 761 (56.4)	
Living alone				
No	29 545 399 (72.3)	4 641 017 (66.7)	1 897 721 (66.0)	<.001
Yes	11 298 610 (27.7)	2 316 482 (33.3)	977 704 (34.0)	
Financial wealth, $, thousands^d^				
Mean (SD)	294.7 (1191.1)	121.1 (514.3)	129.7 (704.0)	<.001
Median (IQR)	25.0 (0.4-165.0)	0.8 (0.0-40.0)	0.3 (0.0-40.0)	<.001
Owns stocks				
No	20 709 641 (50.7)	5 221 529 (75.0)	2 314 313 (80.5)	<.001
Yes	20 134 368 (49.3)	1 735 970 (25.0)	561 112 (19.5)	
Value of stocks if owning stocks, $, thousands				
Mean (SD)	537.5 (1089.7)	404.8 (905.6)	426.8 (703.5)	.15
Median (IQR)	160.0 (40.0-500.0)	125.0 (43.0-400.0)	215.0 (90.0-450.0)	<.001
Owns property				
No	6 978 215 (17.1)	2 238 816 (32.2)	1 302 571 (45.3)	<.001
Yes	33 865 794 (82.9)	4 718 683 (67.8)	1 572 854 (54.7)	
Has ≥1 self-funded retirement account^e^				
No	20 069 292 (49.1)	5 241 528 (75.3)	2 260 696 (78.6)	<.001
Yes	20 774 717 (50.9)	1 715 971 (24.7)	614 729 (21.4)	
Owns ≥1 credit product^f^				
No	24 501 430 (60.0)	5 546 763 (79.7)	2 434 835 (84.7)	<.001
Yes	16 342 579 (40.0)	1 410 736 (20.3)	440 590 (15.3)	
Owns ≥1 risky asset^g^				
No	11 046 884 (27.0)	3 900 440 (56.1)	1 770 837 (61.6)	<.001
Yes	29 797 125 (73.0)	3 057 059 (43.9)	1 104 588 (38.4)	

Abbreviations: CIND, cognitively impaired nondementia; GED, General Educational Development Test; TICS-M, Modified Telephone Interview for Cognitive Status.

^a^ Summary statistics were weighted by Health and Retirement Study analytic weights to represent noninstitutionalized US adults aged 65 years or older in 2018. Numbers of observations are numbers of US older adults represented by the Health and Retirement Study respondents.

^b^ Data are shown as the number (percentage) of individuals unless otherwise indicated.

^c^ Race categories within the Other category were not available in the Health and Retirement Study data owing to small sample sizes and confidentiality concerns. Race information was self-reported and was included in the analysis to illustrate racial disparities in cognitive impairment.

^d^ Financial wealth includes stocks; checking, savings, and money market accounts; certificates of deposit; bonds; and other financial assets less debt. It does not include retirement accounts.

^e^ Self-funded retirement accounts include individual retirement accounts and Keogh plans for unincorporated businesses or self-employed individuals.

^f^ Credit products include mortgages, home equity loans, and home equity lines of credit.

^g^ Risky assets include stocks, self-funded retirement accounts, and credit products.

Most individuals in each cognitive status group, including 7.4 million with CIND or dementia, reported managing finances for the household (Table and Figure). Among those managing finances, 56.6% (95% CI, 51.3%- 62.0%) with dementia and 15.3% (95% CI, 12.8%-17.8%) with CIND reported difficulty doing so compared with 3.8% (95% CI, 3.2%-4.3%) without cognitive impairment (*P* < .001) (Figure); 43.1% of individuals who managed finances with difficulty lived alone. A substantial proportion of respondents with CIND (37.7%; 95% CI, 34.0%-41.3%) or dementia (32.3%; 95% CI, 26.5%-38.1%) who managed finances owned risky assets. Stock accounts had a median value of $215 000 for individuals with dementia and $125 000 for those with CIND (Table).

**Figure.**
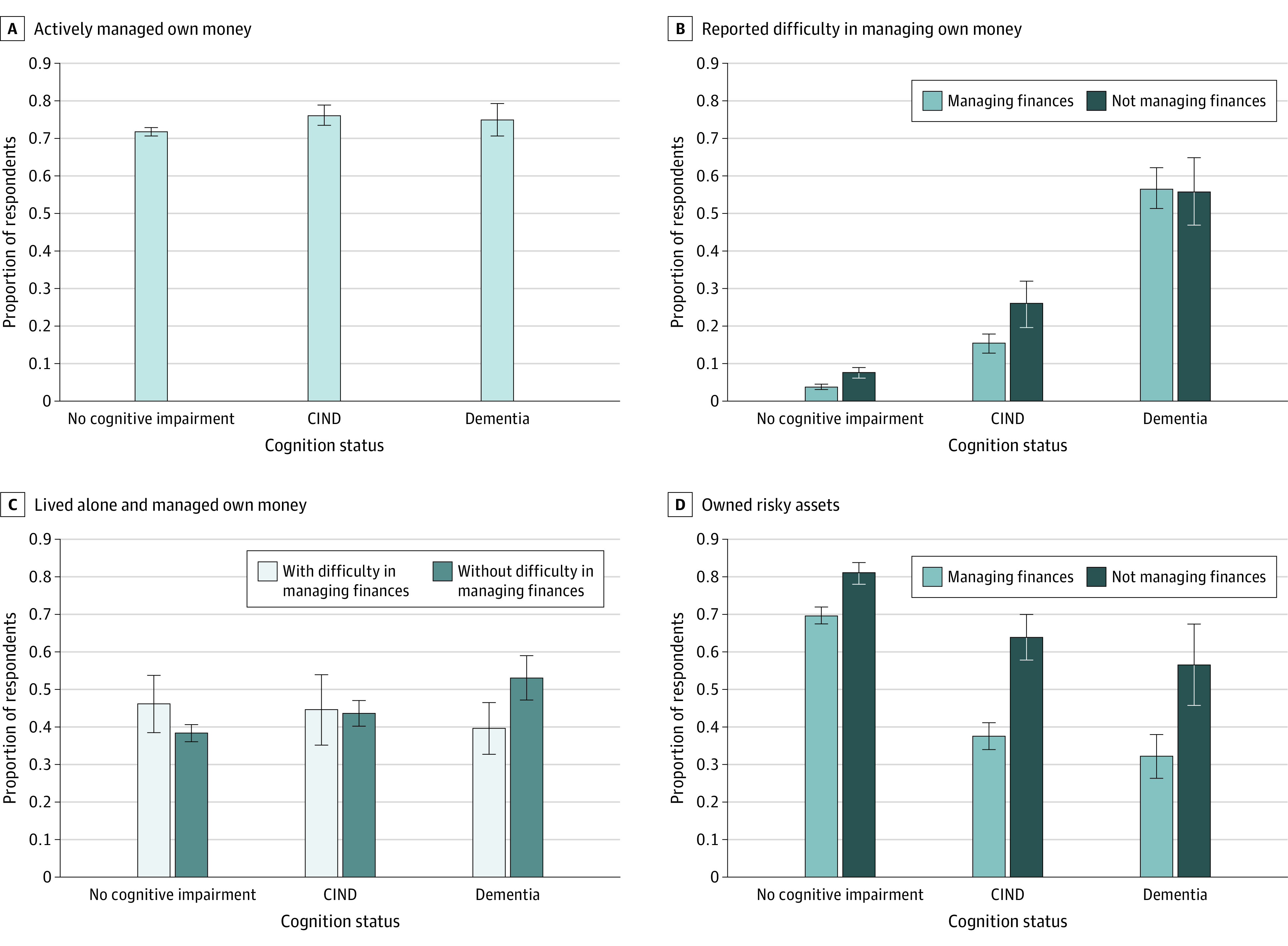
Characteristics of Respondents Managing and Not Managing Their Own Finances by Cognition Status Proportions were weighted by Health and Retirement Study analytic weights to represent noninstitutionalized US adults aged 65 years or older. Whiskers represent 95% CIs. CIND indicates cognitively impaired nondementia.

## Discussion

In 2018, 7.4 million older US adults with dementia or CIND were managing their household finances. Many of these individuals reported difficulty managing their money but owned risky financial assets, which may be particularly susceptible to mismanagement owing to cognitive difficulties.^3,4^ Because cognitive impairment disproportionately affected those who were from racial and ethnic minority groups and individuals with less education, these patterns could further exacerbate socioeconomic disparities. Because of data limitations, we were not able to ascertain clinical dementia diagnosis in the study population or examine more detailed financial behavior such as paying bills.

 Early financial planning, even among cognitively healthy individuals; involvement of extended families or other helpers, especially for those living alone; movement to simpler financial products such as annuities; and availability of financial counseling for households with cognitively impaired members may be useful in management of financial assets.^6^

## Notes

Supplement. **eAppendix 1.** Langa-Weir Classification of Cognitive Function **eAppendix 2.** Sensitivity Analyses Using Alternative Definitions for Cognitive Status Classification
eReferences
