# Supplementary material for: Management of Financial Assets by Older Adults With and Without Dementia or Other Cognitive Impairments
Source: JAMA Netw Open. 2022 Sep 13;5(9):e2231436. doi: 10.1001/jamanetworkopen.2022.31436 (PMC9471975; doi:10.1001/jamanetworkopen.2022.31436)
Supplement: Supplement. — eAppendix 1. Langa-Weir Classification of Cognitive Function eAppendix 2. Sensitivity Analyses Using Alternative Definitions for Cognitive Status Classification eReferences [file jamanetwopen-e2231436-s001.pdf]

Supplementary Online Content

Li J, Wang S, Nicholas LH. Management of financial assets by older adults with and without dementia or other cognitive impairments. *JAMA Netw Open*. 2022;5(9):e2231436. doi:10.1001/jamanetworkopen.2022.31436

eAppendix 1. Langa-Weir Classification of Cognitive Function
eAppendix 2. Sensitivity Analyses Using Alternative Definitions for Cognitive Status Classification
eReferences

This supplementary material has been provided by the authors to give readers additional information about their work.

© 2022 Li J et al. *JAMA Network Open*.

eAppendix 1. Langa-Weir Classification of Cognitive Function

Dementia Ascertainment in Health and Retirement Study (HRS)
There is no standard method of ascertaining cognitive status among the Health and Retirement Study (HRS) respondents. The population-based nature of HRS and the large sample size, which are undoubtedly key advantages of the study design, also makes it infeasible to conduct in-depth clinical assessment needed to accurately diagnose dementia and cognitive impairment for each respondent. Instead, researchers have developed algorithm-based dementia classification methods that leverage the cognitive assessment information collected in HRS, namely the modified version of the Telephone Interview for Cognitive Status (TICS-M).

The Aging, Demographics, and Memory Study (ADAMS)
The Langa-Weir classification of cognitive function[1] is one such algorithm-based classification method that has been developed and validated using the Aging, Demographics, and Memory Study (ADAMS), a substudy of the HRS conducted that involved 3-4 hour in-home neuropsychological and clinical assessments as well as expert clinician adjudication to obtain a gold-standard diagnosis of dementia among a stratified random subsample of about 800 HRS respondents.[2] In ADAMS, individuals are diagnosed as having normal cognitive function, cognitive impairment without dementia (CIND) (defined further below) or dementia using information collected from a detailed neuropsychological assessment and a consensus diagnosis. The cognitive testing in ADAMS included the Mini-Mental State Examination, Boston naming test, digit span (combined forward and backward tasks), Symbol Digit Modality Test, animal fluency, word list three trial learning, construction praxis copying, Trail Making Test, Wechsler Memory Scale, Fuld Object Memory Test, Shipley vocabulary test, and the WRAT 3 blue reading test.[2] ADAMS defines cognitive impairment without dementia (CIND) as "mild cognitive or functional impairment, reported by the participant or informant, or impaired test performance on the neuropsychological measures that does not reach the severity of dementia".[1,3]

Development and Validation of the Langa-Weir Classification of Cognitive Function
The Langa-Weir classification of cognitive function aims to classify all HRS respondents into normal cognitive function, CIND or dementia using information collected from TICS-M administered to all HRS respondents. The TICS-M score ranges from 0-27, with higher scores reflecting better cognitive performance, and is comprised of an immediate and delayed 10-noun free recall test, a serial 7 subtraction test, and a backward count from 20 test.[1]

For HRS respondents who were unable to complete TICS-M due to physical or mental health, a proxy respondent was interviewed instead (when possible) on memory, functional status and behavioral symptoms of the original respondent. For these respondents, the Langa-Weir classification method used the following information collected from proxy respondents to assess cognitive status: a direct assessment of memory ranging from excellent to poor (Score 0–4, with 0 being excellent), an assessment of limitations in five instrumental activities of daily living (IADLs; managing money, taking medication, preparing hot meals, using phones, and doing groceries; Score 0–5, with higher number indicating more limitations). They also used the interviewer assessment of difficulty completing the interview because of cognitive limitation (Score 0–2 indicating, none, some, and prevents completion). These scores were combined to

© 2022 Li J et al. *JAMA Network Open*.

develop a summary score ranging from 0-11, where higher scores reflect worse cognitive performance.[1]

In order to map those scores to cognitive function categories (normal, CIND and dementia), Langa and Weir developed cut-points for the TICS-M score and proxy respondent summary score that would produce the same population distribution of cognitive function categories estimated by ADAMS (i.e., "equipercentile equating").[1] As a result, for TICS-M score, respondents who score from 0 to 6 were classified as demented, 7 to 11 as CIND, and 12 to 27 as normal. Equivalently, for respondents with proxies, those with scores from 6–11 were classified as demented, those with scores from 3–5 as CIND, and 0-2 as normal.

The Langa-Weir is the only method of ascertaining cognitive status in HRS that includes the category of cognitive impairment that is in between normal and dementia (i.e. CIND). Its sensitivity and specificity in dementia ascertainment is shown to be on par with other algorithm-based dementia classification methods.[4] It has also been widely used in prior literature.[5-7]

eReferences

1. Crimmins EM, Kim JK, Langa KM, Weir DR. Assessment of cognition using surveys and neuropsychological assessment: the Health and Retirement Study and the Aging, Demographics, and Memory Study. J Gerontol B Psychol Sci Soc Sci. 2011;66(suppl_1):i162-i171.
2. Langa KM, Plassman BL, Wallace RB, et al. The Aging, Demographics, and Memory Study: study design and methods. Neuroepidemiology. 2005;25(4):181-191.
3. Plassman BL, Langa KM, Fisher GG, Heeringa SG, Weir DR, Ofstedal MB, Burke JR, Hurd MD, Potter GG, Rodgers WL, Steffens DC. Prevalence of cognitive impairment without dementia in the United States. Annals of Internal Medicine. 2008 Mar 18;148(6):427-34.
4. Gianattasio KZ, Wu Q, Glymour MM, Power MC. Comparison of methods for algorithmic classification of dementia status in the health and retirement study. Epidemiology (Cambridge, Mass.). 2019 Mar;30(2):291.
5. Davydow D, Levine D, Zivin K, Katon W, Langa KM. The association of depression, cognitive impairment without dementia, and dementia with risk of ischemic stroke: A cohort study. Psychosomatic Medicine. 2015; 77(2): 200-208.
6. Langa KM, Larson E, Crimmins E, Faul J, Levine D, Kabeto M, Weir D. A comparison of the prevalence of dementia in the United States in 2000 and 2012. JAMA Internal Medicine. 2017; 177(1): 51-58.
7. Wei M, Kabeto M, Langa KM, Mukamal K. Multimorbidity and physical and cognitive function: performance of a new multimorbidity-weighted index. J Gerontol A Biol Sci Med Sci. 2018; 73(2): 225-232.

© 2022 Li J et al. *JAMA Network Open*.

eAppendix 2. Sensitivity Analyses using Alternative Definitions for Cognitive Status Classification

In order to check the robustness of our main study results on management of financial assets by cognitive status, we conducted two sensitivity analyses where we classified HRS respondents' cognitive status using alternative definitions.

1. Alternative cutoff points for non-White respondents

To address the potential concern that racial and ethnic minority respondents might be more likely to receive lower cognitive scores due to either education or language barriers,[1-4] we applied more stringent definitions of dementia and CIND for non-Whites only. Specifically, for non-White respondents, we lowered the cutoff points by 1-pt for both dementia and CIND for non-proxy respondents, and raised the cutoff points by 1-pt for the two categories for proxy respondents. The modified cutoff point for each cognitive status category by subgroup is as follows:

Whites (same as the original Langa-Weir classification):

Cognitive function	Self-respondent (27-point)	Proxy-respondent (11-point)
Whites		
Normal	12-27	0-2
CIND	7-11	3-5
Demented	0-6	6-11

Non-Whites (modified):

Cognitive function	Self-respondent (27-point)	Proxy-respondent (11-point)
Normal	11-27	0-3
CIND	6-10	4-6
Demented	0-5	7-11

We then repeated the entire analyses using the cutoff points above. This sensitivity analysis yielded results that were highly similar to the main study results (omitted but available upon request).

2. Alternative definition of Dementia Incorporating Information on Activities of Daily Living

As a second sensitivity analysis, we used a more stringent definition of (severe) dementia for all HRS respondents,[5] defined as (1) fulfilling the original criteria of dementia using the Langa-Weir classification method, and (2) having limitations in at least three of the six activities of daily living (ADL): walking, dressing, bathing, eating, getting into and out of bed, and toileting. Respondents who satisfied both (1) and (2) were classified as (severe) dementia, and those who satisfied (1) but had fewer than three ADL limitations were combined with those in CIND as the new CIND/mild dementia group. The cognitively normal group remained the same. The results were again consistent with the main study results (available upon request).

© 2022 Li J et al. *JAMA Network Open*.

eReferences

1. Wright CB, DeRosa JT, Moon MP, et al. Race/ethnic disparities in mild cognitive impairment and dementia: the Northern Manhattan Study. J Alzheimers Dis. 2021;80(3):1129-1138.
2. Possin KL, Tsoy E, Windon CC. Perils of race-based norms in cognitive testing: The case of former NFL players. JAMA neurology. 2021;78(4):377-378.
3. Briceño EM, Mehdipanah R, Gonzales XF, et al. Neuropsychological assessment of mild cognitive impairment in Latinx adults: A scoping review. Neuropsychology. 2020;34(5):493.
4. Weuve J, Barnes LL, de Leon CFM, et al. Cognitive aging in black and white Americans: cognition, cognitive decline, and incidence of Alzheimer disease dementia. Epidemiology (Cambridge, Mass). 2018;29(1):151.
5. Nicholas LH, Bynum JP, Iwashyna TJ, Weir DR, Langa KM. Advance directives and nursing home stays associated with less aggressive end-of-life care for patients with severe dementia. Health Affairs. 2014 Apr 1;33(4):667-74.

© 2022 Li J et al. *JAMA Network Open*.
